# RCAN1.4 regulates VEGFR-2 internalisation, cell polarity and migration in human microvascular endothelial cells

**DOI:** 10.1007/s10456-017-9542-0

**Published:** 2017-03-07

**Authors:** Ahmad F. Alghanem, Emma L. Wilkinson, Maxine S. Emmett, Mohammad A. Aljasir, Katherine Holmes, Beverley A. Rothermel, Victoria A. Simms, Victoria L. Heath, Michael J. Cross

**Affiliations:** 1 MRC Centre for Drug Safety Science, Department of Molecular and Clinical Pharmacology, Institute of Translational Medicine, University of Liverpool, Liverpool, L69 3GE UK; 2 Departments of Internal Medicine and Molecular Biology, University of Texas, Southwestern Medical Centre, Dallas, TX USA; 3 Institute of Cardiovascular Sciences, The Medical School, University of Birmingham, Birmingham, B15 2TT UK; 4 King Abdullah International Medical Research Center (KAIMRC)-Eastern Region, King Saud bin Abdulaziz University for Health Sciences, Al Hasa, 31982 Saudi Arabia

**Keywords:** VEGFR-2, RCAN1, Endothelial, Polarisation, Migration, Angiogenesis

## Abstract

Regulator of calcineurin 1 (RCAN1) is an endogenous inhibitor of the calcineurin pathway in cells. It is expressed as two isoforms in vertebrates: RCAN1.1 is constitutively expressed in most tissues, whereas transcription of RCAN1.4 is induced by several stimuli that activate the calcineurin-NFAT pathway. RCAN1.4 is highly upregulated in response to VEGF in human endothelial cells in contrast to RCAN1.1 and is essential for efficient endothelial cell migration and tubular morphogenesis. Here, we show that RCAN1.4 has a role in the regulation of agonist-stimulated VEGFR-2 internalisation and establishment of endothelial cell polarity. siRNA-mediated gene silencing revealed that RCAN1 plays a vital role in regulating VEGF-mediated cytoskeletal reorganisation and directed cell migration and sprouting angiogenesis. Adenoviral-mediated overexpression of RCAN1.4 resulted in increased endothelial cell migration. Antisense-mediated morpholino silencing of the zebrafish RCAN1.4 orthologue revealed a disrupted vascular development further confirming a role for the RCAN1.4 isoform in regulating vascular endothelial cell physiology. Our data suggest that RCAN1.4 plays a novel role in regulating endothelial cell migration by establishing endothelial cell polarity in response to VEGF.

**Electronic supplementary material:**

The online version of this article (doi:10.1007/s10456-017-9542-0) contains supplementary material, which is available to authorized users.

## Introduction

Angiogenesis is the process via which new blood vessels are formed from pre-existing vessels. Physiological angiogenesis is critical for normal physiological development [1], whilst pathological angiogenesis plays a role in the development of inflammatory diseases, cancer and diabetic retinopathy [2–4]. Vascular endothelial growth factor A (VEGF) is a potent pro-angiogenic regulator which is secreted by tissues in response to hypoxia or inflammation [5].

VEGF signalling in vascular endothelial cells is primarily mediated by VEGF receptor 2 (VEGFR-2) [6]. The binding of VEGF to VEGFR-2 results in receptor activation, characterised by receptor autophosphorylation and initiation of a series of signalling cascades within the cell resulting in migration, cell survival and proliferation [6–8]. Phosphorylation of the tyrosine 1175 residue within the intracellular C-terminal domain of the receptor induces activation of phospholipase C-γ (PLCγ) [9], which hydrolyses phosphatidylinositol (4,5)-bisphosphate (PIP_2_), to generate inositol (1,4,5)-trisphosphate (IP_3_) and diacylglycerol (DAG). IP_3_ acts upon receptors on the endoplasmic reticulum (ER) to induce the release of intracellular stores of Ca^2+^. This increase in Ca^2+^ induces activation of the protein phosphatase calcineurin (PP2B) [10]. Dephosphorylation of nuclear factor of activated T-cells (NFAT) by calcineurin results in its translocation to the nucleus, where, in cooperation with other transcription factors, it induces the transcription of a number of VEGF-responsive genes important for regulating endothelial cell physiology [11, 12].

Regulator of calcineurin 1 (RCAN1) belongs to a family of endogenous negative regulators of calcineurin action [13]. Originally named Down syndrome critical region 1 (DSCR1), the human RCAN1 gene is located on the 21q22.1-q22.2 region of chromosome 21 and is expressed primarily in brain, heart and skeletal muscle [14] and in endocrine tissues such as the adrenal gland [15] and pancreas [16]. The RCAN1 gene is composed of seven exons and alternative splicing generates two isoforms: RCAN1.1 (252 amino acids, 39 kDa) and RCAN1.4, (192 amino acids; 29 kDa) which have identical C-terminal domains but differ at their N-termini due to the actions of alternative promoters, leading to the inclusion of either exon 1 or exon 4, respectively [14]. RCAN1.1 is constitutively expressed in most tissues, whereas transcription of RCAN1.4 is induced by several stimuli that activate the calcineurin-NFAT pathway [17, 18]. The role of RCAN1 in vivo has been studied in RCAN1^−/−^ mice where gene ablation of RCAN1.1 and RCAN1.4 resulted in mice with no anatomical differences [19]. However, another study has shown that vascular contraction is reduced in RCAN1^−/−^ mice suggesting that RCAN1 may regulate vascular function [20]. A more recent study with RCAN1^−/−^ mice focussing on tumour angiogenesis reported hyperactivation of the calcineurin/NFAT pathway in endothelial cells from these mice, resulting in endothelial cell apoptosis. This led to decreased tumour angiogenesis in a human tumour xenograft study [21]. RCAN1.4 was identified as a VEGF-responsive gene in endothelial cells [20, 22, 23] and has been shown to negatively regulate VEGF-mediated gene expression [14, 24] and regulate VEGF-mediated endothelial cell migration [24, 25].

In this study, we were interested in determining the mechanism through which RCAN1.4 regulates endothelial cell migration. We have utilised siRNA-mediated silencing of RCAN1 and adenoviral-mediated overexpression of RCAN1.1 and RCAN1.4 splice variants to reveal that RCAN1.4 is able to drive the directed migration of endothelial cells via a transient interaction with VEGFR-2. The ability of RCAN1.4 to regulate vascular formation in vivo was identified in zebrafish embryos.

## Materials and methods

### Materials

Human recombinant VEGF-A_165_ and HGF were obtained from Peprotech. Fibroblast growth factor-2 (FGF-2) was obtained from R&D Systems. All other materials were obtained from Invitrogen unless otherwise stated.

### Antibodies

Antibodies against phospho-VEGFR-2 (Y1173, #3770), phospho-AKT (S473, #4058), phospho-ERK1/2 (T202/Y204, #9101), VEGFR-2 (#2479), phospho-HGFR/c-Met (Y1349; #3121) transferrin receptor (TfR) (#13113) and GAPDH (#5174) were purchased from Cell Signalling Technology (New England Biolabs). The antibody against RCAN1 (#D6694) was purchased from Sigma. Antibody against GM130 (#610823) was purchased from BD Bioscience. The antibody against pericentrin (#ab4448) was purchased from Abcam. Antibodies against VEGFR-2 (N-terminal extracellular domain; #AF357), HGFR/c-Met (N-terminal extracellular domain; #AF276) and calcineurin-A (#MAB2839) were purchased from R&D Systems.

### Cell culture

HDMEC (#C-12210; juvenile) were purchased from Promocell and were cultured in endothelial cell basal media (EBM) MV2 growth media (C-22221; Promocell), supplemented with 5% (v/v) foetal calf serum (FCS) and EGF (5 ng/ml), VEGF-A (0.5 ng/ml), FGF-2 (10 ng/ml), long r3 insulin growth factor-1 (20 ng/ml), hydrocortisone (0.2 µg/ml) and ascorbic acid (1 µg/ml) (supplement pack C-39221; Promocell). Cells were routinely cultured on 0.5% (w/v) gelatin-coated plates in a humidified incubator under 5% CO_2_ at 37 °C. HEK 293 cells were cultured in Dulbecco’s modified Eagle’s medium (DMEM) supplemented with 10% (v/v) FCS. For growth factor stimulation, cells were serum starved for at least 16 h by changing media to media supplemented with 1% (v/v) FCS without growth factor supplements. Growth factors were used for the times indicated at 50 ng/ml.

### siRNA transfection

The following siRNA duplexes were obtained from Qiagen: RCAN1 (Hs_DSCR1_5 HP, SI03224900; Hs_DSCR1_6 HP, SI03246208) and VEGFR-2 (Hs_KDR_5, SI00605528; Hs_KDR_6, SI00605535). siRNA duplexes were used at a final total concentration of 10 nM in 0.1% (v/v) Lipofectamine RNAiMAX, according to the manufacturer’s instructions. Control non-silencing (N.S.) transfections were performed using all-stars Negative Control siRNA (Qiagen) at a concentration of 10 nM. Transfection reactions were performed in serum-free OptiMEM (Life Technologies). Cell media were changed to serum containing media 6 h after transfection and cells were left for a further 48 h prior to experiments.

### Adenoviral overexpression

HDMECs were plated at 50,000 cells/well in 12 well plates. Cells were grown for 24 h in the plates and infected with an empty control virus (Ad-Control) or adenovirus encoding human RCAN1.1 (Ad-RCAN1.1) and RCAN1.4 (Ad-RCAN1.4) [26] at a multiplicity of infection (MOI) of 50. After 6 h, the virus was removed and the media were changed to 1% (v/v) FCS EBM for 20 h before stimulation with growth factors.

### Western immunoblotting

Cells were harvested in ice-cold lysis buffer (20 mM Tris pH7.5, 150 mM NaCl, 2.5 mM EDTA, 10% (w/v) glycerol and 1% (w/v) Triton X-100, 1 mM Na_3_VO_4_ (Sigma #S-6508), 10 µg/ml Aprotinin (Sigma #A-4529), 10 µg/ml Leupeptin (Sigma #L-8511), 10 µg/ml Pepstatin, 1 mM PMSF) and kept on ice with gentle agitation for 15 min to allow complete lysis. The cells were scraped into 1.5 ml Eppendorf tubes and cleared of cell debris by centrifugation at 17,000 ×*g* for 20 min at 4 °C and the supernatant transferred to fresh tubes. Total solubilised protein was measured using the BCA method (Pierce). For SDS-PAGE an appropriate volume of 1 × LDS (Life Technology #NP0008) sample loading buffer was added to the sample (10 μg of protein), which was then heated at 90 °C for 5 min before loading onto pre-cast 4-12% Bis–Tris NuPAGE gels (Thermo Fisher Scientific). Proteins were separated using MOPS SDS running buffer (Thermo Fisher Scientific) for 2 h at 200 V and 50 mA. Proteins were transferred to nitrocellulose (Hybond C, GE Healthcare) and membranes blocked in 5% (w/v) BSA. Blots were probed with primary antibody in 2% (w/v) BSA followed by secondary HRP-coupled antibody, after several washes. Washed membranes were incubated in enhanced chemiluminescence substrate (RPN2106; GE Healthcare) and developed using Fuji Medical X-Ray Film, Super RX, 100NF (Jet X-Ray, UK). The immune reactive bands detected on X-ray film were quantified by scanning (Bio Rad #GS-800, Photoshop CS6) and densitometric analysis achieved using ImageJ (National Institute of Health (NIH), Version 1.47n). GAPDH levels were quantified to correct for protein loading.

### Immunoprecipitation

Cells were seeded on gelatin-coated dishes in normal growth media for 24 h. Adenovirus was added to cells for 6 h. Media were changed to 1% (v/v) FCS-containing media for 20 h before stimulation with VEGF (50 ng/ml) for 0, 2, 10 and 30 min. Cells were harvested in ice-cold lysis buffer (20 mM Tris pH7.5, 150 mM NaCl, 2.5 mM EDTA, 10% (w/v) glycerol and 1% (w/v) Triton X-100, 1 mM Na3VO4 (Sigma #S-6508), 10 µg/ml Aprotinin (Sigma #A-4529), 10 µg/ml Leupeptin (Sigma #L-8511), 10 µg/ml Pepstatin, 1 mM PMSF) and kept on ice with gentle agitation for 15 min to allow complete lysis. The cells were scraped into 1.5 ml Eppendorf tubes and cleared of cell debris by centrifugation at 17,000 ×*g* for 20 min at 4 °C and the supernatant transferred to fresh tubes. Lysates were incubated with anti-RCAN1 antibody (25 µg/mL) or control rabbit IgG (25 µg/mL, Millipore #12-370) rotating end over end for 20 h at 4 °C. Protein G Sepharose was added to this for a further 3 h before samples were centrifuged at 10,000 × g for 1 min and washed three times with lysis buffer. Supernatant was removed and the samples resuspended in LDS sample buffer before boiling at 90 °C for 5 min. Lysates were resolved by SDS-PAGE gels followed by the western blotting protocol. For IP experiments, Goat anti-Rabbit IgG-HRP Fc-specific (#111-035-046, Jackson Labs) was used for detecting RCAN1 Ab.

### Cell surface biotinylation and internalisation

HDMECs were plated in 6 cm dishes in normal growth media for 48 h. Media were then changed to 1% (v/v) FCS-containing media for 24 h. Cell monolayers were washed in PBS containing Mg^2+^ and Ca^2+^ and incubated with sulfo-NHS-SS-biotin (0.5 mg/ml) (Thermo Fisher Scientific) for 30 min at 4 °C. Following surface labelling, cells were washed in PBS and incubated with endothelial media containing 1% FCS v/v. Cells were stimulated with VEGF (50 ng/ml) for 10 min. Following stimulation and receptor internalisation, cells were then transferred onto ice and washed twice with ice-cold PBS and biotin removed from proteins remaining at the cell surface by reduction with 100 mM 2-mercaptoethanesulphonate (MesNa; Sigma) in 50 mM Tris, 100 mM NaCl (pH 8.6) for 2 × 10 min at 4 °C. To evaluate efficiency of biotin cleavage, control cells were kept in PBS without MesNa during this step. Unreacted MesNa was quenched with 20 mM iodoacetamide (Sigma) for 10 min and the cells lysed in ice-cold lysis buffer as above. Pre-cleared lysates were incubated with streptavidin coupled agarose at 4 °C and mixed by end-over-end rotation at 4 °C for 3 h. Beads were centrifuged at 17,000 × *g* for 1 min and washed × 3 in lysis buffer and resuspended in 50 μl of 2 × LDS sample buffer. Samples were analysed by SDS-PAGE before immunoblotting for VEGFR-2 and TfR.

### Immunofluorescence imaging

Cells were plated on gelatin-coated glass coverslips. Following agonist stimulation, coverslips were washed twice in PBS and fixed with 2% (w/v) PFA for 15 min at room temperature. Cells were quenched in PBS containing 50 mM ammonium chloride and then permeabilised in PBS containing 0.2% (w/v) Triton X-100 for 10 min at room temperature. Cells were washed in PBS and then blocked for 1 h at room temperature with TBS containing 0.1% (v/v) Tween-20 and 5% (w/v) BSA. Coverslips were incubated with primary antibody in TBS containing 0.1% (v/v) Tween-20 and 1% (w/v) BSA. Cells were washed × 5 in TBST and then incubated with the relevant secondary Alexa Fluor coupled antibodies for 1 h at room temperature. Samples were then incubated for 10 min with Hoechst 33342 (Invitrogen #H-21492) and mounted with Prolong gold (Invitrogen). Fluorescent images were acquired with a Zeiss AxioObserver inverted fluorescence microscope with Apotome2 using a Plan-Apochromat 40x/1.3 oil immersion objective and Zeiss ZenPro software.

### Scratch wound migration assay

HDMECs were seeded in a 12-well plate in normal growth media for 48 h. Media were then changed to 1% (v/v) FCS-containing media for 24 h and a scratch introduced into the cell monolayer using a sterile 200 μl pipette tip. Cells were stimulated with growth factor for 18 h before washing with PBS then fixed with 2% (w/v) PFA for 15 min at room temperature. The scratch was visualised using a Nikon Eclipse TS100 inverted light microscope (10× objective) fitted with LCD colour imaging system. To calculate cell migration, a time zero image was taken immediately after scratch wounding the cells. After 18 h, a further three images were taken of each scratch and migration measured in each image to give an average value for each condition compared to the time zero point.

### Time-lapse chemotaxis assays

HDMECs were seeded in a 12-well plate in normal growth media for 48 h. Media were then changed to 1% (v/v) FCS-containing media for 24 h and a scratch introduced into the cell monolayer using a sterile 200 μl pipette tip. Cells were stimulated with growth factor, and migration of individual cells was recorded at 5 min intervals with an Axiovert 200 M inverted microscope with atmosphere control unit for 18 h using a 20× objective and digital video recording. Migration trajectories were tracked for 20 individual cells over 20 h using Ibidi chemotaxis and migration tool.

### Cell polarisation

Confluent monolayers of HDMECs were grown on gelatin-coated coverslips and serum starved for 20 h. A horizontal wound was created in the confluent monolayer using a sterile 200 µl pipette tip and cells stimulated with VEGF (50 ng/ml) for 18 h. Subsequently, cells were fixed in 2% (w/v) PFA in PBS for 10 min and permeabilised in 0.25% (w/v) Triton X-100 in PBS for 5 min followed by 1 h blocking in 2% (w/v) BSA in PBS at room temperature. After blocking, cells were incubated for 1 h in primary antibody staining Golgi (GM130; 1:100 in 2% (w/v) BSA in PBS) and centrosomes (Pericentrin; 1:100 in 2% (w/v) BSA in PBS). Cells were subjected to an additional incubation of 1 h with a mixture of a fluorescently labelled Alexa Fluor secondary antibody and Hoechst 33342 at room temperature. Cells were washed at least three times between each step. Following mounting, images were captured using an inverted Zeiss AxioObserver inverted fluorescence microscope with Plan-Apochromat 20x/0.8 and Zeiss ZenPro software. The polarity index was determined as previously described [27]. Briefly, cells in which the Golgi and centrosome was located within the 120 °C angle facing the major axis of the wound were scored as polarised. A total of 100 cells from each of three independent experiments were analysed to determine the polarity index.

### Proximity ligation assay (PLA)

HDMECs were seeded on gelatin-coated glass coverslips in normal growth media for 48 h. Media were then changed to 1% (v/v) FCS-containing media for 24 h and a scratch introduced into the cell monolayer using a sterile 200 μl pipette tip. Cells were allowed to recover for 1 h prior to agonist stimulation for the required time. Cells were fixed in 4% (w/v) PFA on ice for 10 min and permeabilised in PBS containing 0.1% (v/v) Triton X-100 for 20 min and thereafter subjected to in situ PLA using the Duolink Detection kit (Olink Bioscience, Uppsala, Sweden) according to the manufacturer’s instructions for Duolink Blocking solution and Detection protocol. Briefly, slides were blocked, incubated with antibodies directed against VEGFR-2 (R&D, Goat anti-human VEGFR-2 #AF357) and RCAN1 (Sigma, Rabbit anti-RCAN1, #D6694) and thereafter incubated with relevant PLA probes conjugated to unique oligonucleotides. Circularisation and ligation of the oligonucleotides were followed by an amplification step. The products were detected by a complementary fluorescently labelled probe, and slides were mounted using Vectashield (Vector Laboratories Inc, Burlingame, CA) and evaluated using a Zeiss AxioObserver inverted fluorescence microscope. Z-stack micrographs were taken with the Plan-Apochromat 40x/1.3 oil immersion objective and Zeiss ZenPro software. The number of proximity ligations, visualised as bright fluorescent signals, was counted in 20 fields/well for cells at the leading edge and also for cells not at the edge in the main body. Quantifications are given as mean ± s.e.m.

## 3D in vitro sprouting angiogenesis assay

A sprouting angiogenesis assay was performed as described [28]. HDMEC were grown on gelatin-coated dishes. Four hours after siRNA transfection, cells were labelled with either 5 μM cell tracker green CMFDA (Invitrogen #C2925) or 5 μM cell tracker orange CMRA (Invitrogen #C34551) for 45 min. Labelled HDMECs were detached by accutase and mixed equally 1:1 to give a final cell number of 1000 and incubated with 0.20% (w/v) carboxymethylcellulose and seeded in ultra-low adherent (ULA) round‐bottom 96‐well plates (Corning) and grown overnight. 16‐24 h later, spheroids were collected and embedded into 200 μl of collagen I gel in glass bottom dishes (Greiner). After polymerisation, spheroids were cultured for 24 h in the presence of EBM2 medium containing 1% (v/v) FCS and VEGF (50 ng/ml). Cells were fixed in 2% (w/v) PFA for 10 min and sprouting angiogenesis analysed by immunofluorescence using a Plan-Apochromat 20x/0.8 and Zeiss ZenPro software. The number of tip and stalk cells of each colour were counted. Experiments were repeated reversing the cell tracker dyes to ensure no dye bias.

### Zebrafish analysis

The transgenic line of zebrafish TG(fli1:GFP) [29] were bred to produce embryos. Embryos were microinjected at the 1–2 cell stage with 5 ng of either mismatch or morpholino oligonucleotide solutions (MOs) [30]. Co-injection of phenol red was performed to act as a tracer for the injection procedure. The morpholino sequences were RCAN1a-4: ACTTCATTGTTTTCAGGTGCATGAC and mismatch control: ACaTgATTcTTTTgAGcTGCATGAC (Gene Tools, Oregon, USA). Vasculature imaging at 48 hpf was performed using a Zeiss 780 Zen confocal microscope. Computer analysis of the vasculature was performed using ImageJ and the following plugins: Skeletonise (2D/3D) and Analyze Skeleton plugins.

### Statistical analysis

Statistical analysis was performed using an unpaired Student’s *t* test in Excel. *P* values <0.05 were considered significant. *P < 0.05, **P < 0.01, ***P < 0.001.

## Results

### RCAN1 regulates VEGFR-2 internalisation following ligand stimulation

Human RCAN1 comprises seven exons, which are alternatively spliced resulting in two major protein isoforms RCAN1.1 (252 amino acids) and RCAN1.4 (197 amino acids). The proximal promoter regulating expression of RCAN1.4 is activated by calcineurin-NFAT signalling in response to different stimuli such as VEGF [22, 23]. We have previously shown that in endothelial cells, VEGF stimulates an increase in RCAN1.4 expression but not RCAN1.1 [24]. We subsequently showed that RCAN1 was required for VEGF/VEGFR-2 mediated migration of endothelial cells. In the current study, we were interested in determining the mechanism by which RCAN1 regulates VEGFR-2 function and endothelial cell migration.

**Fig. 1:**
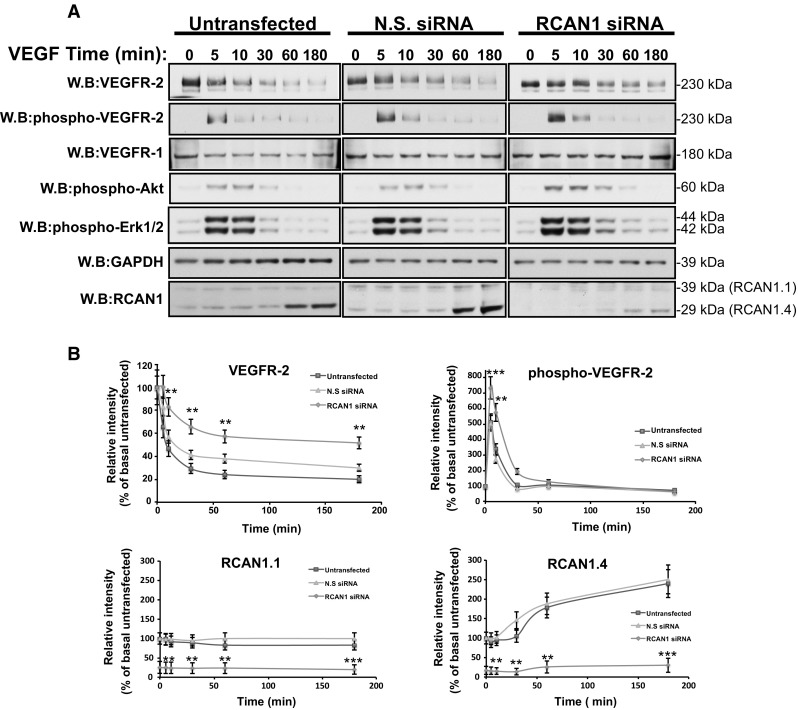
RCAN1 regulates VEGFR-2 levels in HDMECs. **a** Endothelial cells were left untransfected or transfected with non-silencing siRNA (N.S. siRNA) or RCAN1 siRNA. Cells were left unstimulated or stimulated with VEGF165 (50 ng/ml) for a range of time (5, 10, 30, 60, 180 min). Cells were lysed and immunoblotted with antibodies to VEGFR-1, phospho-VEGFR-2 (Y1175), VEGFR-1, phospho-AKT (S473), phospho-ERK1/2 (T202/Y204), GAPDH and RCAN1. Results are from one experiment representative of three separate experiments. **b** Quantification of levels of VEGFR-2, phospho-VEGFR-2, and RCAN1.1 and RCAN1.4. Levels are calculated relative to % of basal untransfected (mean ± s.e.m. *n* = 3 independent experiments). *P < 0.05, ** P < 0.01, ***P < 0.001 (unpaired student’s t test comparing RCAN1 siRNA and N.S. siRNA)

Ligand binding to VEGFR-2 results in activation of an intracellular signalling cascade and ubiquitination and receptor internalisation followed by proteolytic degradation [31]. In HDMECs, VEGF stimulation led to a transient increase in VEGFR-2 phosphorylation and time-dependent decrease in total VEGFR-2 levels (Fig. 1a, b). To analyse downstream signalling pathways, we measured AKT phosphorylation, which is required for endothelial cell survival and ERK1/2 phosphorylation, required for cell proliferation [6]. VEGF stimulation resulted in a transient increase in AKT phosphorylation and ERK1/2 phosphorylation. VEGF stimulation also resulted in an increase in RCAN1.4 expression after 30–60 min with no apparent increase in RCAN1.1 levels (Fig. 1a, b). In order to analyse the role of RCAN1 in VEGFR-2 phosphorylation and downstream signalling, we utilised short-interfering RNA (siRNA)-mediated gene silencing. The duplexes utilised target both isoforms of RCAN1 (RCAN1.1 and RCAN1.4) as it was not possible to design isoform specific duplexes. siRNA-mediated silencing of RCAN1 resulted in a delayed VEGFR-2 downregulation following VEGF stimulation with a concomitant increase in receptor phosphorylation (Fig. 1a, b).

**Fig. 2:**
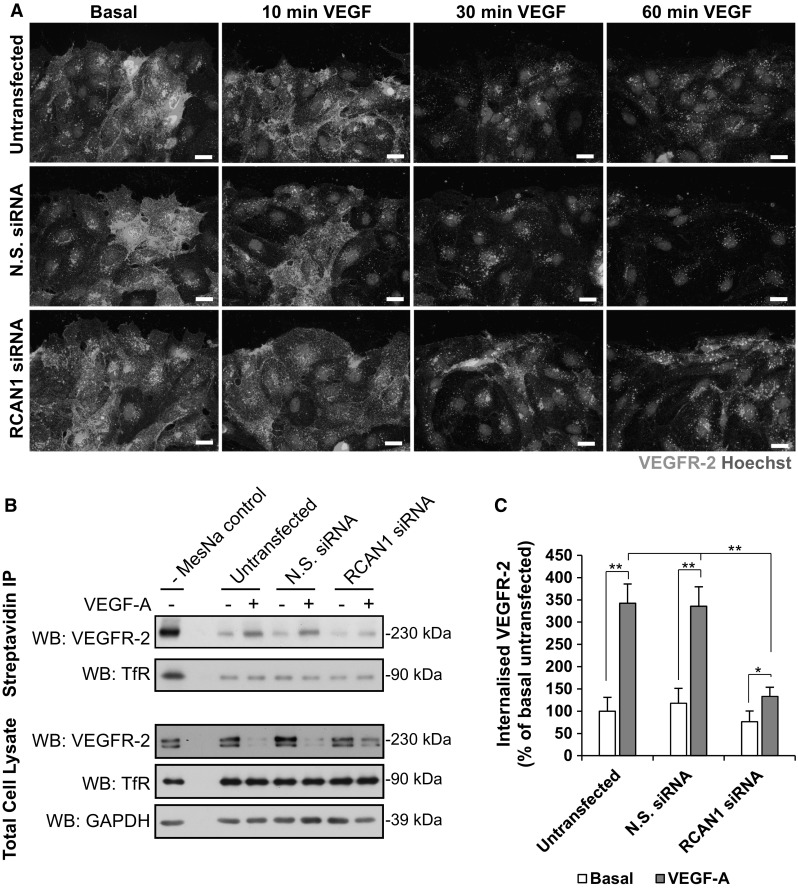
RCAN1 regulates VEGFR-2 internalisation.** a** HDMECs were left untransfected or transfected with either non-silencing siRNA (N.S. siRNA) or RCAN1 siRNA. Cells were stimulated with VEGF (50 ng/ml) for 10, 30 and 60 min. Cells were fixed in 2% PFA and left unpermeabilised. Cells were incubated with goat anti-VEGFR-2 antibody (recognising the N-terminal, extracellular domain) followed by incubation with donkey anti-goat Alexa488 antibody. Nuclei were stained with Hoechst 33342. Scale bar represents 20 μm.** b** HDMECs were left untransfected or transfected with non-silencing siRNA (N.S.siRNA) or RCAN1 siRNA. Cells were surface labelled with cleavable biotin and stimulated or not with VEGF (50 ng/ml) for 10 min. Biotin remaining on the surface was cleaved with MesNa and biotinylated proteins immunoprecipitated with streptavidin-agarose. Internalised VEGFR-2 and transferrin receptor (TfR) were detected by Western blotting. Efficiency of biotin cleavage was analysed in untransfected cells without MesNa. The level of VEGFR-2, TfR and GAPDH was analysed in total cell lysate.** c** Quantification of internalised VEGFR-2 (mean ± S.D. from three individual experiments). *P < 0.05, **P < 0.01, (unpaired student’s t test)

In order to analyse cell surface VEGFR-2 levels, we utilised an antibody to the external N-terminal domain of VEGFR-2 and performed immunofluorescence analysis following ligand stimulation in the absence of permeabilisation. Stimulation with VEGF resulted in a time-dependent decrease in surface VEGFR-2 levels in HDMECs, an effect substantially reduced following siRNA-mediated silencing of RCAN1 (Fig. 2a). To confirm this effect on cell surface VEGFR-2, we utilised cell surface labelling with cleavable biotin. Following stimulation with VEGF, VEGFR-2 that has undergone internalisation from the membrane is protected from biotin cleavage and can be specifically analysed in cell lysates by immunoprecipitation with streptavidin-agarose. This method confirmed that VEGF stimulation resulted in VEGFR-2 internalisation at 10 min, an effect that was substantially reduced by silencing of RCAN1 (Fig. 2b, c). Analysis of the internalisation of the transferrin receptor (TfR), which undergoes constitutive internalisation and recycling in cells [32, 33], revealed that silencing of RCAN1 did not affect internalisation of the TfR (Fig. 2b).

In order to see if this effect of RCAN1 silencing on receptor levels and downstream signalling was apparent with another RTK expressed on HDMECs, we analysed the levels of the hepatocyte growth factor receptor (HGFR/c-Met) following stimulation with HGF. Analysis of HGFR by western blotting revealed that RCAN1 silencing did not appear to affect HGFR levels (Supplementary Fig. 1a, b). Further analysis by immunofluorescence using an antibody to the extracellular N-terminal domain of HGFR in the absence of cell permeabilisation revealed no apparent effect of RCAN1 silencing on HGF-stimulated HGFR internalisation (Supplementary Fig. 2).

### RCAN1 regulates VEGFR-2-mediated endothelial cell polarity and cytoskeletal reorganisation

**Fig. 3:**
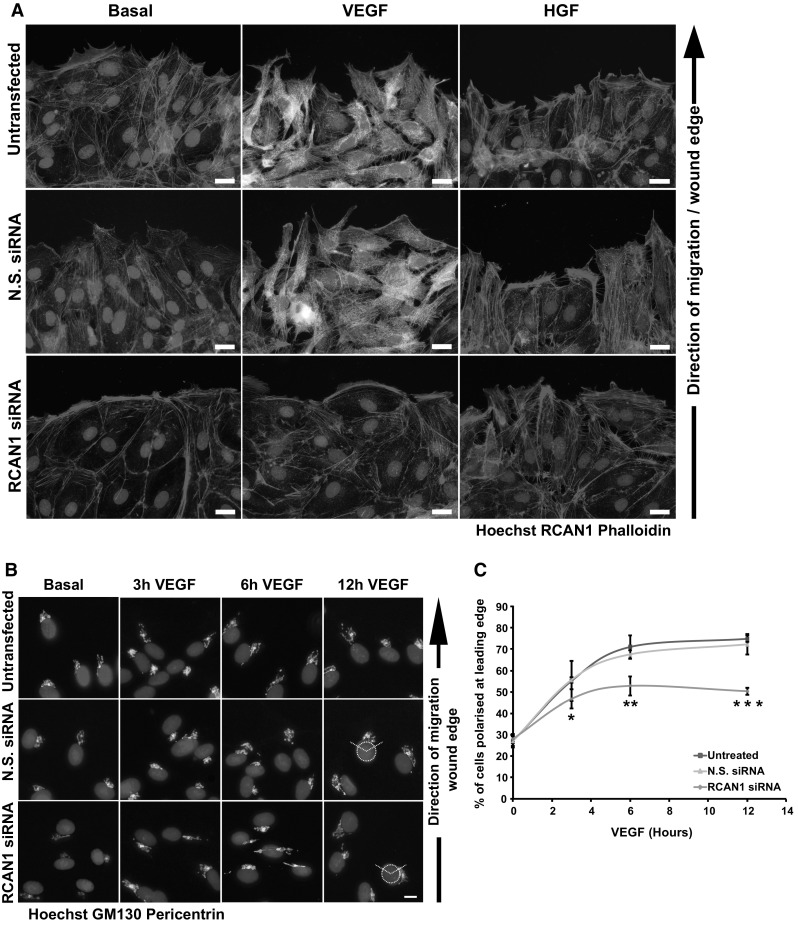
RCAN1 regulates VEGF-mediated cytoskeletal reorganisation and cell polarity. **a** HDMECs were grown on gelatin-coated glass coverslips. Cells were left untransfected or transfected with either non-silencing siRNA (N.S. siRNA) or RCAN1 siRNA. A horizontal wound was introduced on the glass coverslip. Cells were stimulated or not with either VEGF (50 ng/ml) or HGF (50 ng/ml) for 3 h. Cells were fixed in 2% PFA, permeabilised and stained for RCAN1. Cytoskeletal reorganisation was visualised by staining F-actin with phalloidin-Alexa Fluor 588. Nuclei were stained with Hoechst 33342. Scale bar represents 20 μm. **b** HDMECs were grown on gelatin-coated glass coverslips. Cells were left untransfected or transfected with either control non-silencing siRNA (N.S. siRNA) or RCAN1 siRNA. A horizontal wound was introduced on the glass coverslip. Cells were stimulated or not VEGF (50 ng/ml) for 3, 6 and 12 h. Cells were fixed in 2% PFA, permeabilised and stained for golgi (GM130) and centrosomes (pericentrin). Cell polarisation towards the wound was assessed by drawing a circle centred over the nucleus with a 120° segment facing the wound. Cells were polarised if the golgi and centrosome were located within this 120° segment. Scale bar represents 20 μm. **c** Quantification of cell polarisation. The percentage of polarised cells was calculated by counting 100 cells per treatment. Results are plotted as % of cells polarised at the leading edge (mean ± s.e.m. n = 3 independent experiments). *P < 0.05, **P < 0.01, ***P < 0.001

Directed cell migration in response to agonists requires asymmetric activation of cell membrane receptors to induce polarised signals that result in the generation of protrusions at the front of the cell (leading edge) and retraction at the back of the cell (trailing edge) [34, 35]. Analysis of the cytoskeletal reorganisation at the scratch wound edge in HDMECs revealed that VEGF induced a number of cellular protrusions reminiscent of filopodia (Fig. 3a). This effect was blocked by siRNA-mediated silencing of RCAN1 expression as leading edge cells were devoid of any protrusions under both basal and VEGF-stimulated conditions. However, HGF, which does not induce RCAN1.4 levels (Supplementary Fig. 1a), was able to induce cytoskeletal reorganisation even in HDMECs with depleted RCAN1 levels, revealing that RCAN1 did not block cytoskeletal reorganisation per se (Fig. 3a). The scratch wound assay allows analysis of cell polarity, as directed migration in this assay is accompanied by reorganisation of the centrosome and Golgi apparatus, relative to the nucleus, to face the direction of cell migration [36, 37]. To analyse the effect of RCAN1 silencing on HDMEC cell polarity, we visualised the orientation of the Golgi using an antibody to GM130 and the centrosome using an antibody to pericentrin. Following VEGF stimulation of HDMECs cell polarity increased at the leading edge over a period of 6 h from 28 ± 2 to 70 ± 5%. This increase in cell polarity was considerably reduced in RCAN1-depleted cells, achieving a maximum of only 50 ± 5% (Fig. 3b).

### RCAN1.4 regulates VEGFR-2 dependent endothelial cell migration

**Fig. 4:**
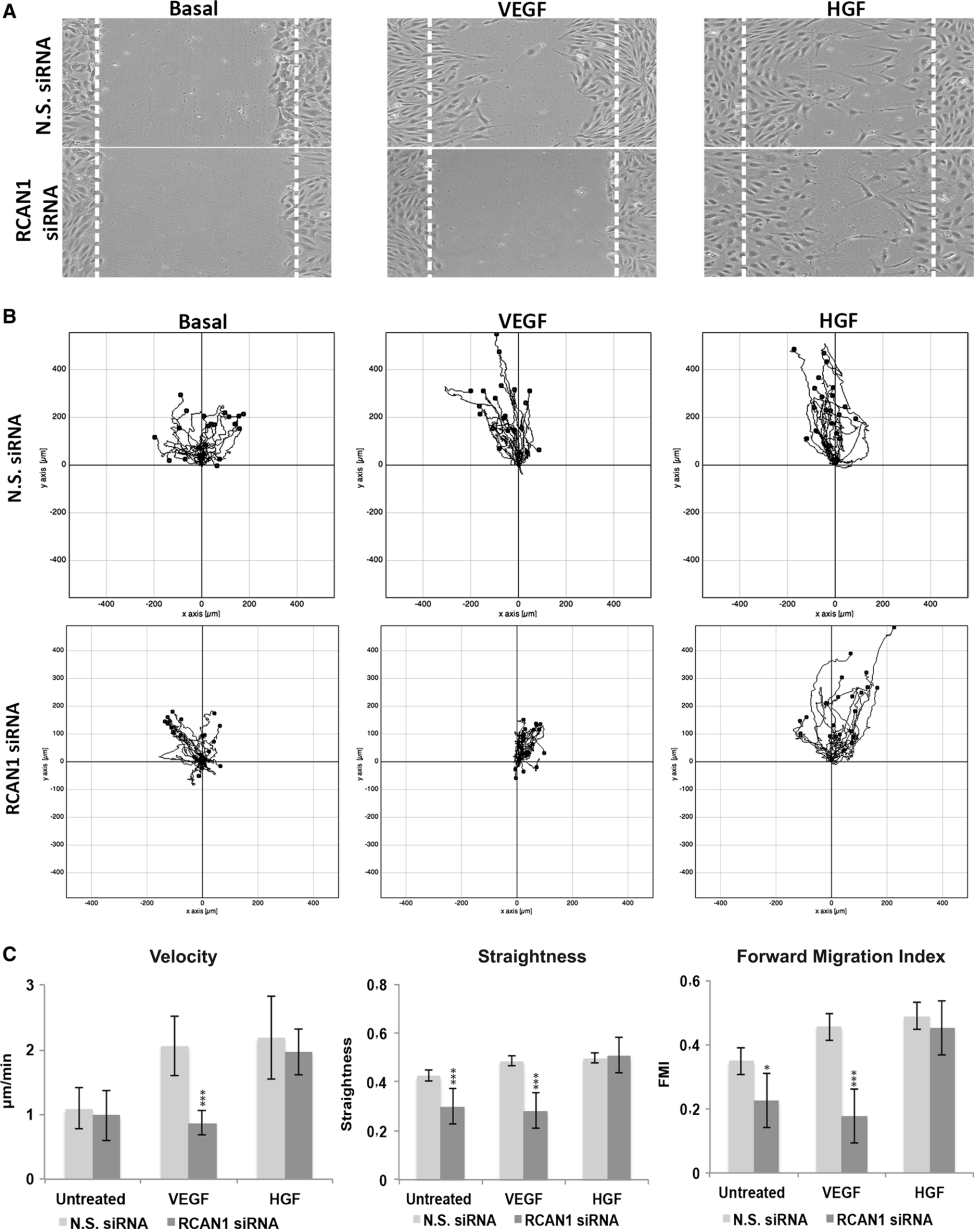
RCAN1 regulates VEGF-mediated directional cell migration. HDMECs were plated in gelatin-coated 24-well plates and transfected with either non-silencing siRNA (N.S. siRNA) or RCAN1 siRNA prior to incubation in media containing 1% FCS for 24 h. A horizontal scratch wound was introduced. Cell were stimulated or not with either VEGF_165_ (50 ng/ml) or HGF (50 ng/ml). Low-magnification (4× objective) phase contrast time-lapse series were obtained (images collected every 15 min for 18 h).** a** Images of cells following 18 h stimulation.** b** Migration trajectories of representative cells (n = 20). Tracks of cells migrating in a representative field of imaging.** c** Comparison of velocity (displacement/time), straightness (displacement/total path length) and forward migration index (FMI) (mean ± S.D., n = 3). *P < 0.05, ***P < 0.001 (unpaired student’s t test)

**Fig. 5:**
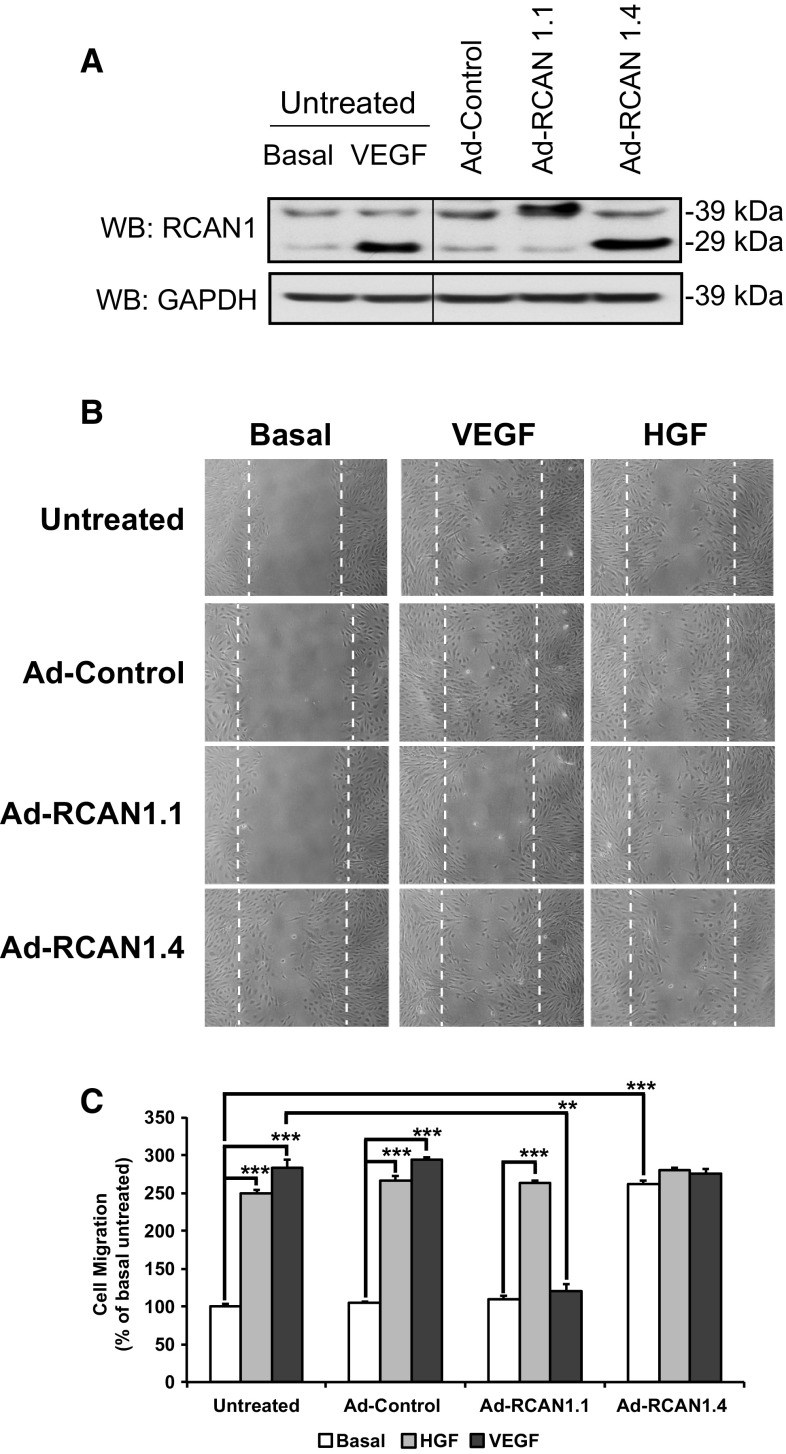
RCAN1.4 isoform stimulates endothelial cell migration. HDMECs were plated on gelatin-coated 24-well plates and incubated with adenovirus (Ad-Control) or adenovirus encoding RCAN1.1 isoform (Ad-RCAN1.1) or RCAN1.4 isoform (Ad-RCAN1.4) for 24 h.** a** Cells were lysed and western blotted for levels of RCAN1.1 (39 kDa) and RCAN1.4 (29 kDa) and GAPDH. VEGF stimulation (50 ng/ml for 1 h) of untransfected cells were analysed for levels of RCAN1.4 as a control.** b** Cells were incubated in media containing 1% FCS for 24 h and a horizontal scratch wound was introduced. Cells were incubated with media (Basal) or stimulated with either VEGF (50 ng/ml) or HGF (50 ng/ml) for 18 h. Cells were fixed in 2% PFA.** c** Quantification of cell migration. Results are plotted as cell migration, % of basal untreated response (mean ± S.D., n = 3). ***P < 0.001 (unpaired student’s t test)

Our previous studies have identified a role for RCAN1.4 in regulating VEGF-mediated migration of endothelial cells using a scratch wound assay [24]. We were interested in determining the specificity of RCAN1.4 in regulating VEGF function. RCAN1 silencing specifically inhibited cell migration in response to VEGF, but not HGF (Fig. 4a). The finding that RCAN1 depletion causes a defect in cell migration led us to examine the directionality of migration. We utilised time-lapse DIC imaging of HDMEC following scratch wounding and agonist stimulation with VEGF or HGF. Migration trajectories were tracked for 20 individual cells over 20 h using the Ibidi chemotaxis and migration tool. RCAN1 depletion resulted in a slower and more convoluted migration in response to VEGF compared to untransfected and non-silencing conditions (Fig. 4b). In contrast, RCAN1 depletion did not affect HGF-stimulated cells, which showed normal distance and trajectory indicating that RCAN1 silencing did not block migration *per se*. Similarly, the velocity (total path length/time), straightness (displacement/total path length) and forward migration index [38] were significantly decreased for the RCAN1-depleted cells stimulated with VEGF in contrast to HGF (Fig. 4c). We have previously shown that RCAN1.4 levels are sustained for at least 9 h in HDMECs following VEGF stimulation, with no change in RCAN1.1 levels [24]. To confirm that RCAN1.4 was able to regulate VEGFR-2-mediated migration in endothelial cells, we utilised adenovirus-mediated gene transduction to specifically over-express the RCAN1.1 and RCAN1.4 isoforms in HDMECs (Fig. 5a); levels of overexpression of RCAN1.4 were similar to levels induced by VEGF stimulation in untreated cells. Infection with adenovirus encoding RCAN1.4 resulted in an increase in HDMEC migration similar to that evoked by VEGF (Fig. 5b, c). In contrast, infection with adenovirus encoding RCAN1.1 did not stimulate migration and inhibited VEGF-mediated migration, with no apparent effect on HGF-stimulated migration (Fig. 5b, c).

**Fig. 6:**
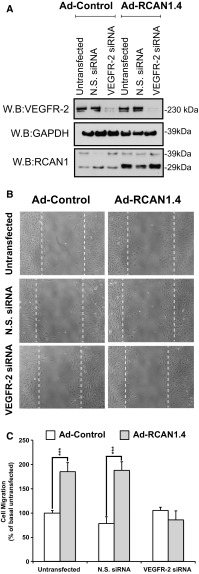
RCAN1.4 stimulated cell migration requires VEGFR-2.** a** HDMECs were plated on gelatin-coated plates and transfected with either non-silencing siRNA (N.S. siRNA) or KDR siRNA prior to incubation with adenovirus (Ad-Control) or adenovirus encoding RCAN1.4 (Ad-RCAN1.4). Cells were lysed and immunoblotted with antibodies to VEGFR-2, GAPDH and RCAN1.** b** Cells were incubated with siRNA and adenovirus as above before a horizontal wound was introduced to the well. Cells were fixed in 2% PFA.** c** Results are plotted as cell migration, % of basal untreated response (mean ± S.D., n = 3). ***P < 0.001 (unpaired student’s t test)

We were interested in determining if the effect of over-expression of RCAN1.4 on HDMEC migration was dependent on VEGFR-2. To address this, we utilised siRNA-mediated gene silencing to significantly reduce VEGFR-2 expression in HDMECs (Fig. 6a). Cell migration stimulated by RCAN1.4 overexpression was prevented in the cells treated with VEGFR-2 siRNA suggesting that RCAN1.4-mediated cell migration required VEGFR-2 (Fig. 6b, c).

### RCAN1.4 transiently co-localises and binds with VEGFR-2

**Fig. 7:**
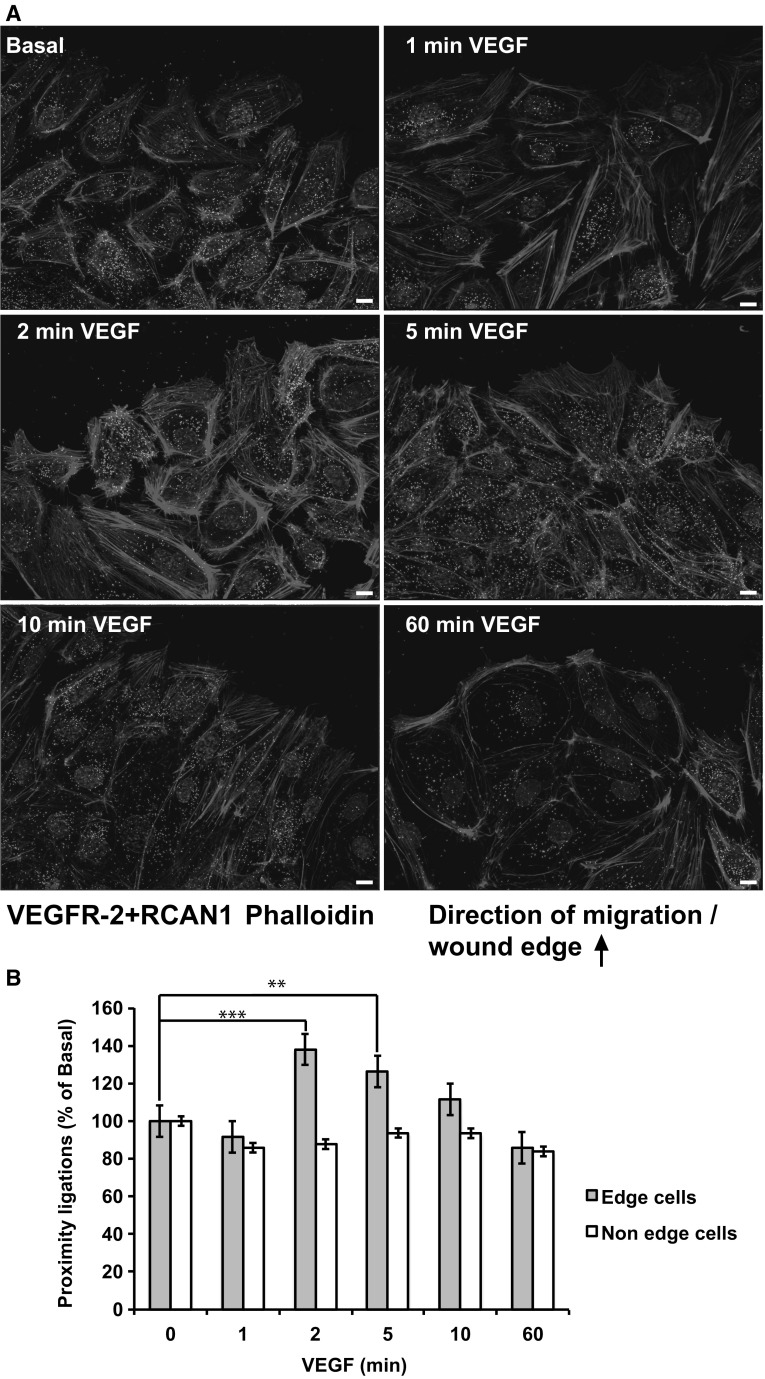
RCAN1 and VEGFR-2 interaction at the leading edge of migrating cells.** a** HDMECs were plated on gelatin-coated glass coverslips. A horizontal wound was introduced on the glass coverslip. Cells were stimulated or not with VEGF (50 ng/ml) for 1, 2, 5, 10 and 60 min. Cells were fixed in 2% PFA, permeabilised and stained for interaction between RCAN1 and VEGFR-2 using proximity ligation reaction (PLA) with antibodies to RCAN1 and VEGFR-2. Scale bar represents 20 μm.** b** Quantification of RCAN1/VEGFR-2 proximity ligations. Cells were analysed at the leading edge and also in the main body with the number of proximity ligations counted for 100 cells (mean ± s.e.m., n = 3 independent experiments). **P < 0.01, ***P < 0.001

**Fig. 8:**
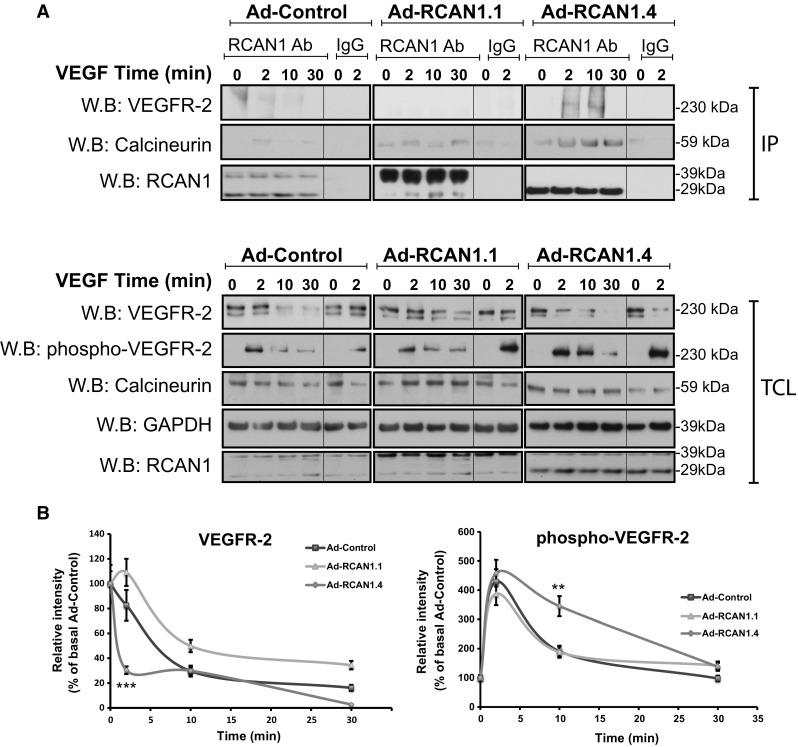
RCAN1.4 binds to VEGFR-2.** a** HDMECs were plated on gelatin-coated dishes and incubated with adenovirus (Ad-Control) or adenovirus encoding RCAN1.1 (Ad-RCAN1.1) or RCAN1.4 (Ad-RCAN1.4) isoforms. Cells were stimulated with VEGF (50 ng/ml) for 0, 2, 10 and 30 min. Cells were lysed and immunoprecipitation (IP) was performed using antibodies to RCAN1 or control rabbit IgG. Proteins were separated by SDS-PAGE and immunoblotted with antibodies to phospho-VEGFR-2 (Y1175), VEGFR2, calcineurin-A, GAPDH and RCAN1. Total cell lysate (TCL) was also analysed by Western blotting.** b** Quantification of levels of VEGFR-2 and phospho-VEGFR-2 in TCL. Levels are calculated relative to % of basal Ad-Control (mean ± s.e.m. n = 3 independent experiments). **P < 0.01, ***P < 0.001 (unpaired student’s t test)

The profound effect of RCAN1 depletion on delaying internalisation of ligand-activated VEGFR-2 suggested that RCAN1 may interact directly with VEGFR-2. We utilised a proximity ligation assay (PLA) to analyse any potential spatial and temporal interaction between VEGFR-2 and RCAN1. This technique generates a fluorescence signal at the precise site of interaction of the proximity ligation probes and consequently the primary antibodies targeting RCAN1 and VEGFR-2 [39]. The PLA revealed a rapid and transient increased co-localisation between VEGFR-2 and RCAN1 in the HDMECs at the leading edge following stimulation with VEGF (Fig. 7a, b). In order to explore the potential association between RCAN1 and VEGFR-2 further we overexpressed both the RCAN1.1 and RCAN1.4 variants in HDMECs using adenoviral-mediated gene transduction. Immunoprecipitation of RCAN1 revealed that only the RCAN1.4 variant was capable of transiently interacting with VEGFR-2 (Fig. 8a). However, both RCAN1 variants were capable of interacting with calcineurin, a known binding partner of RCAN1 proteins [40]. Analysis of total cell lysate (TCL) revealed that overexpression of RCAN1.4 resulted in a transient increase in VEGF-mediated downregulation of VEGFR-2 levels compared with cells expressing Ad-RCAN1.1 and Ad-Control (Fig. 8a, b).

### RCAN1.4 regulates sprouting angiogenesis and vascular development in zebrafish embryos

**Fig. 9:**
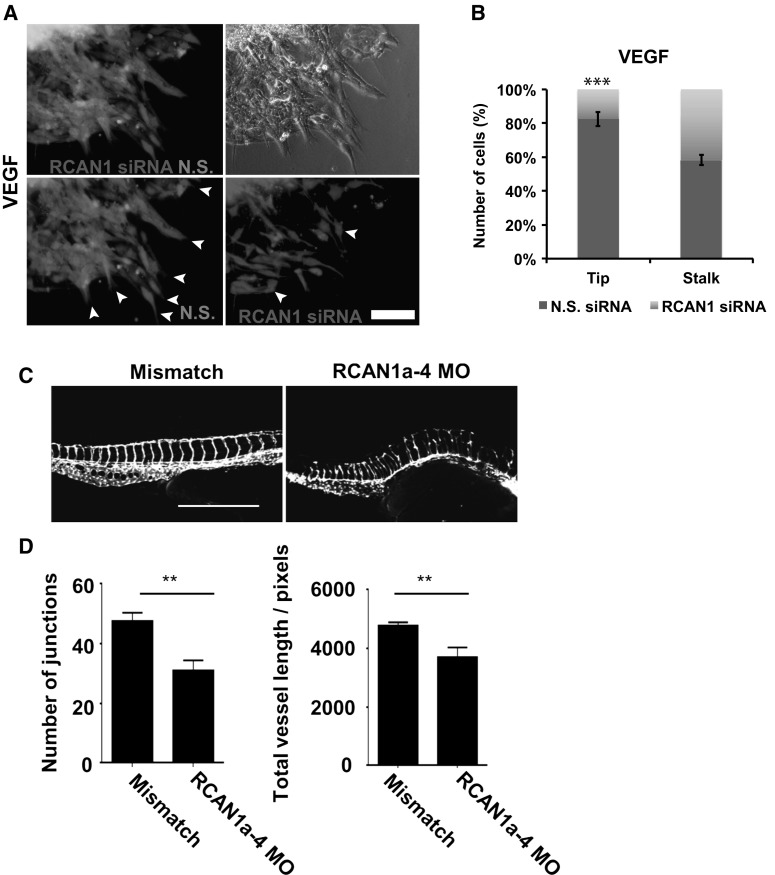
RCAN1.4 regulates endothelial cell sprouting and vascular formation in zebrafish.** a** In vitro sprouting angiogenesis assay. HDMECs were transfected with control non-targeting siRNA (N.S. siRNA) or RCAN1 siRNA prior to incubation with cell tracker dye green or orange. Cells were mixed 1:1 and incubated with carboxymethycellulose in ULA 96-well plates for 18 h prior to embedding in collagen matrix. Cells were stimulated with VEGF (50 ng/ml) for 18 h and fixed in 2% PFA prior to fluorescence imaging.** b** Quantification of endothelial tip and stalk cell formation. Sprouting tubes from VEGF treated spheroids were quantified (mean ± S.D., n = 3). ***P < 0.001 (unpaired Student’s t test).** c** Transgenic fli1-GFP zebrafish embryos were injected with mismatch or RCAN1a-4 morpholino oligonucleotides and imaged at 48 hpf. Scale bar represents 500 μm.** d** Analyses of total vessel length and junction number, n = 14 for mismatch, n = 15 for RCAN morpholino. Asterisk indicates P < 0.01 (unpaired student’s t test)

Sprouting angiogenesis requires endothelial tip cells to become polarised and extend filopodia with the coordinated regulation of stalk cells, allowing the formation and stabilisation of the sprout and ultimately a lumen-containing vessel [41]. We have previously shown that RCAN1 is required for efficient tubular morphogenesis using a collagen gel assay [24]. To investigate the role of RCAN1 in vascular sprouting we generated 3D endothelial cell spheroids composed of a 1:1 mixture of non-silencing control and RCAN1 siRNA silenced HDMECs labelled with different fluorescent dyes and embedded these in a collagen gel. RCAN1 depleted HDMECs showed a reduced ability to achieve a tip position compared to control cells when stimulated with VEGF (Fig. 9a, b).

The role of RCAN1.4 in angiogenesis in vivo was investigated in zebrafish, using the transgenic Fli1-GFP fish in which endothelial cells constitutively express GFP [29]. Translation of the zebrafish RCAN1.4 orthologue (RCAN1a-4) was blocked by micro-injection of an antisense morpholino targeting the translational start site of this splice variant; as a control a mismatch morpholino was used. The fish embryos were imaged at 48 h post-fertilisation and the images analysed to determine total vessel length and junction number. Knockdown resulted in a disrupted vascular network with incomplete sprouting of the intersomitic vessels and defective fusion at the dorsal lateral anastomosing vessel (Fig. 9c). Analyses of the images determined that there was a significant decrease in the total vessel length and junction number in the morpholino knockdown embryos (Fig. 9d). These data indicate that RCAN1.4 plays a role in vascular development in zebrafish.

## Discussion

We have previously reported the novel finding that in addition to its role in inhibiting calcineurin function and subsequently VEGF-stimulated gene expression, RCAN1.4 is able to regulate VEGF-mediated tubular morphogenesis in endothelial cells [24]. Our present study was designed to determine the mechanism through which RCAN1.4 is able to regulate VEGFR-2 signalling and affect endothelial cell function. We now report the novel finding that RCAN1.4 is able to specifically regulate VEGFR-2 internalisation and subsequently cell polarisation leading to efficient directed cell migration and vascular sprout formation.

Our previous work has shown that VEGF is unique, compared with other growth factors such as FGF and HGF, in stimulating RCAN1.4 expression in HDMECs via a pathway requiring calcineurin and PKC-delta [24]. VEGF-mediated upregulation of RCAN1.4 has been previously thought to act as a negative feedback loop in preventing calcineurin-mediated gene expression in endothelial cells and tempering sustained VEGFR-2 activity. Use of siRNA targeting both the RCAN1.1 and RCAN1.4 isoforms defined a role for RCAN1 in regulating VEGFR-2 internalisation (Fig. 1,  2a), VEGF-mediated cytoskeletal reorganisation (Fig. 3a), cell polarisation (Fig. 3b) and cell migration (Fig. 4). Our data are in agreement with a previous study using antisense oligonucleotides to RCAN1 which reported an inhibition of VEGF-mediated migration in human umbilical vein endothelial cells (HUVECs) [25].

Overexpression of each RCAN1 isoform by use of adenoviral-mediated gene transduction revealed that whilst RCAN1.4 could stimulate endothelial cell migration, in the absence of VEGF, RCAN1.1 did not stimulate migration but instead inhibited VEGF-induced migration with no effect on HGF-stimulated migration (Fig. 5b, c). This is in agreement with a previous report showing that lentiviral-mediated overexpression of RCAN1.1 inhibited VEGF-mediated migration of HUVECs [42]. Taken together, this data suggest that RCAN1 isoforms have contrasting functions in regulating VEGF-mediated migration in endothelial cells.

The ability of RCAN1.4 to regulate VEGF-mediated migration required the presence of VEGFR-2 (Fig. 6a–c) suggesting a potential interaction between RCAN1.4 and VEGFR-2. RTK internalisation follows the canonical clathrin-dependent endocytic route involving the small GTP binding protein Rab5 [43, 44]. Analysis of the intracellular interaction with VEGFR-2 and RCAN1 by proximity ligation assay (PLA) showed a close association between VEGFR-2 and RCAN1, which transiently increased at the leading edge following VEGF stimulation (Fig. 7). Immunoprecipitation revealed that whilst both RCAN1.1 and RCAN1.4 can interact with calcineurin, in agreement with previous findings [40], only RCAN1.4 was able to transiently bind to VEGFR-2 (Fig. 8a). Both RCAN1 proteins are able to inhibit calcineurin activity by binding via their common c-terminal tail to the catalytic site on calcineurin [40]. Taken together, this would suggest that the unique N-terminal region of RCAN1.4 (Exon 4) transiently interacts with VEGFR-2 at the leading edge of cells. The lack of effect of siRNA-mediated silencing of RCAN1 expression on TfR internalisation (Fig. 2b) and agonist-stimulated HGFR/c-Met internalisation (Supplementary Fig. 2) suggests that RCAN1.4 effects on receptor internalisation may be specific to VEGFR-2 in endothelial cells. Analysis of the amino acid sequence of RCAN1.4 reveals no classical SH2 or SH3 domains required for association with RTKs, suggesting that RCAN1.4 may interact with VEGFR-2 by a novel mechanism, possibly directly with the VEGFR-2 or via ancillary proteins specifically required for VEGFR-2 internalisation, such as neuropilin 1 (Nrp-1) [45], synectin [46] and the recently described ephrin–B2 [47].

It is also possible that RCAN1.4 can influence VEGFR-2 phosphorylation, as RCAN1 depletion resulted in decreased VEGFR-2 degradation and increased transient phosphorylation on Y1175 (Fig. 1a). However, overexpression of RCAN1.4 did not appear to affect phosphorylation of VEGFR-2 under basal conditions but did appear to transiently enhance VEGF-stimulated phosphorylation of VEGFR-2 (Fig. 8a). Previous data have shown that VEGFR-2 internalisation is required for phosphorylation of Y1175 in VEGFR-2 in mouse endothelial cells [47]. Dephosphorylation of VEGFR-2 is regulated by a number of recently identified phosphatases such as VE-PTP [11], which causes delayed VEGFR-2 internalisation [48]. However, other data have shown that VEGFR-2 internalisation is not required for VEGF-mediated phosphorylation in HUVECs [49]. Our data suggest that whilst RCAN1.4 levels can regulate VEGFR-2 internalisation and proteolysis, effects on receptor phosphorylation are not as marked. Studies on guided cell migration in *Drosophila* have revealed the physiological role of RTK endocytosis is to ensure localised intracellular response to guidance cues by stimulating spatial restriction of signalling allowing organised cell migration [50]. Recent data have shown that VEGFR-2 endocytosis regulates endothelial sprout formation [51]. Our finding that RCAN1.4 regulates cell polarity and cytoskeletal reorganisation at the leading edge suggests that RCAN1.4 facilitates the coupling of VEGFR-2 internalisation to generation of cell polarity at the leading edge of a scratch wound and in tip cells in an angiogenic sprout. In zebrafish, there are two orthologues of RCAN1, RCAN1a and RCAN1b, but only the former encodes orthologues of the alternatively spliced 1.1 and 1.4 isoforms (Ensemble genome browser). In this study, translation of RCAN1a-4 was specifically blocked giving rise to defective vessel development, indicating that the function of this splice variant is conserved across species.

The role of RCAN1 in vivo has been studied in RCAN1^−*/*−^ mice where gene ablation of RCAN1.1 and RCAN1.4 resulted in mice with no anatomical differences indicating that RCAN1 is not essential for development [19]. However, the vasculature was not studied in detail and it is possible that these mice may have more subtle changes in their vasculature. It is also possible that other members of the RCAN gene family compensate for the loss of RCAN1 [52]. Interestingly, a role for RCAN1 in neurite outgrowth and guidance has been recently identified [53]. Neurons from RCAN1^−/−^ mice fail to orient towards brain-derived neurotrophic factor (BDNF), whereas transgenic RCAN1 neurons exhibit enhanced outgrowth. This data suggest that TrkB, the receptor for BDNF, and VEGFR-2 may share a common requirement for RCAN1 proteins to regulate neuronal cell and endothelial cell guidance, respectively.

**Fig. 10:**
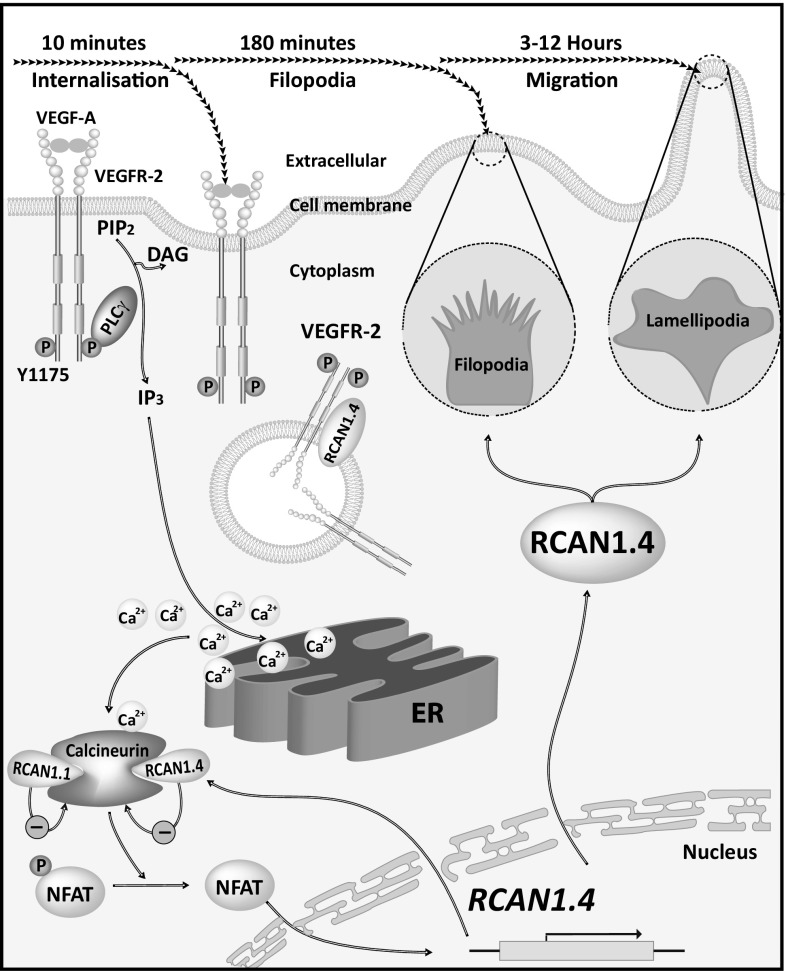
Proposed mechanism for the role of RCAN1.4 in regulating VEGF action on endothelial cells. VEGF binding to VEGFR-2 leads to autophosphorylation on a number of c-terminal tyrosine residues resulting in the activation of PLC-γ and cleavage of PtdIns(4,5)P_2_ (PIP_2_) generating diacylglycerol (DAG) and Ins(1,4,5)P_3_ (IP_3_). Following VEGFR-2 phosphorylation, RCAN1.4 transiently interacts with VEGFR-2 to regulate internalisation at the leading edge. The resulting Ins(1,4,5)P_3_-mediated release of Ca^2+^ from the endoplasmic reticulum (ER) results in activation of calcineurin/PP2B and dephosphorylation of the nuclear factor activated T-cells (NFAT) transcription factor which translocates to the nucleus and increases transcription of RCAN1.4 mRNA. The resulting increase in RCAN1.4 protein acts on calcineurin to decrease NFAT-mediated gene transcription. Increased RCAN1.4 protein also acts independently of calcineurin to regulate VEGFR-2-mediated cytoskeletal reorganisation, filopodia and lamellipodia formation resulting in endothelial cell polarisation and directed migration

Our data describe a novel role for RCAN1.4 in regulating VEGFR-2 internalisation and directed cell migration (Fig. 10). Our current knowledge of RCAN1.4 is based on its discovery as an endogenous competitive inhibitor of calcineurin. This interaction with the catalytic domain of calcineurin is mediated via the exon 7 encoded c-terminus of RCAN1.4 [54] and leads to downregulation of NFAT-mediated inflammatory genes [22, 24]. Our data define a novel role for RCAN1.4, in regulating VEGFR-2 internalisation and endothelial cell migration.

## Electronic supplementary material

Below is the link to the electronic supplementary material.

Supplementary material 1 (DOCX 11 kb)

Supplementary material 2 (EPS 4074 kb)

Supplementary material 3 (EPS 15057 kb)
