# Supplementary material for: RCAN1.4 regulates VEGFR-2 internalisation, cell polarity and migration in human microvascular endothelial cells
Source: Angiogenesis. 2017 Mar 7;20(3):341–58. doi: 10.1007/s10456-017-9542-0 (PMC5511620; doi:10.1007/s10456-017-9542-0)
Supplement: Supplementary file 1 — Supplementary material 1 (DOCX 11 kb) [file 10456_2017_9542_MOESM1_ESM.docx]

**Supplementary figure legends.**

**Supplementary Figure 1. RCAN1 does not regulate HGFR levels in HDMECs.** (A). HDMECs were left untransfected or transfected with non-silencing siRNA (N.S. siRNA) or RCAN1 siRNA. Cells were left unstimulated or stimulated with HGF (50 ng/ml) for a range of time (5,10,30,60,180 min). Cells were lysed and immunoblotted with antibodies to HGFR (c-Met), phospho-HGFR (Y1349), GAPDH and RCAN1. Results are from one experiment representative of 3 separate experiments. (B) Quantification of levels of HGFR, phospho-HGFR (Y1349), RCAN1.1 and RCAN1.4. Levels are calculated relative to % of basal untransfected from one experiment representative of three. * P<0.05, ** P<0.01, *** P<0.001 (unpaired student’s t-test comparing RCAN1 siRNA and N.S. siRNA).

**Supplementary Figure 2. RCAN1 does not regulate HGFR internalisation in HDMECs.** HDMECs were left untransfected or transfected with either non-silencing siRNA (N.S. siRNA) or RCAN1 siRNA. Cells were stimulated with HGF (50 ng/ml) for 10, 30 or 60 min. Cells were fixed in 2% PFA and left unpermeabilised. Cells were incubated with goat anti-HGFR/c-Met antibody (recognising the N-terminal, extracellular domain) followed by incubation with donkey anti-goat Alexa488 antibody. Nuclei were stained with Hoechst 33342. Scale bar represents 20 μm.
